# Belumosudil for Long‐Standing Refractory Chronic Graft‐Versus‐Host Disease: A Two‐Patient Case Report

**DOI:** 10.1002/ccr3.73050

**Published:** 2026-06-25

**Authors:** Mihee Kim, Ho Cheol Jang, Ga‐Young Song, Sung‐Hoon Jung, Seo‐Yeon Ahn, Jae‐Sook Ahn

**Affiliations:** ^1^ Hematology–Oncology Chonnam National University Hwasun Hospital Hwasun‐gun Jeollanam‐do Republic of Korea

**Keywords:** belumosudil, case report, chronic graft‐versus‐host disease, fibrotic cGVHD, patient‐reported outcomes, ROCK2 inhibitor

## Abstract

In long‐standing fibrotic chronic GVHD refractory to multiple systemic therapies, belumosudil may provide clinically meaningful patient‐reported improvement and reduce infection‐related hospitalizations even when early NIH organ scores remain stable. When benefits are evident and safety is acceptable, continuation beyond 3–6 months may be reasonable.

## 1 Introduction

Chronic graft‐versus‐host disease (cGVHD) is a major late complication of allogeneic hematopoietic stem cell transplantation (allo‐HSCT) and a leading cause of long‐term morbidity and nonrelapse mortality [1, 2]. Its phenotype is heterogeneous, with fibrotic features across multiple organ systems—including cutaneous, pulmonary, fascial/joint, ocular/oral mucosal, hepatobiliary, gastrointestinal, and genital domains—to varying degrees. Even in the era of modern GVHD prophylaxis, including post‐transplant cyclophosphamide‐based approaches, clinically significant chronic GVHD remains a major cause of late morbidity. Its phenotype is highly heterogeneous, encompassing fibrotic manifestations across multiple organ systems—including the skin, lungs, fascia and joints, ocular and oral mucosa, hepatobiliary tract, gastrointestinal tract, and genital organs—to varying degrees [3]. These manifestations are often difficult to reverse and may become refractory after multiple lines of therapy [4, 5, 6]. Systemic corticosteroids remain first‐line treatment, yet durable control is frequently elusive and cumulative toxicity is common; only a subset of patients can discontinue systemic therapy, and failure‐free survival after initial therapy remains limited [1, 7].

Fibrosis in cGVHD reflects persistent allo/autoimmune activation and dysregulated immune–stromal crosstalk that drive myofibroblast differentiation and extracellular matrix deposition. Hallmark mechanisms include skewing toward Th17/Tfh (T helper 17/T follicular helper) responses with relative regulatory T‐cell deficiency, aberrant germinal center B‐cell activity, and profibrotic cytokine signaling (e.g., interleukin 17/interleukin 21, transforming growth factor β), culminating in progressive sclerosis of skin and fascia and small‐airway obstruction, among other organ systems [2, 6, 8, 9, 10]. These immune–fibrotic circuits provide a rationale for therapies that modulate both inflammation and fibrogenesis [4, 5, 6, 10].

Rho‐associated coiled‐coil–containing protein kinase 2 (ROCK2) is a nodal regulator of T‐cell differentiation and profibrotic transcriptional programs; its inhibition attenuates Th17/Tfh pathways, favors regulatory T‐cell function, and downregulates profibrotic gene expression [11]. Belumosudil, an oral, selective ROCK2 inhibitor, is approved for patients with cGVHD after at least two prior systemic therapies and has demonstrated organ‐level activity and symptom improvement across prospective studies (including the phase 2 ROCKstar trial) and subsequent clinical reports [8, 9, 12, 13, 14].

Here, we describe two cases of multi‐line persrefractory, long‐standing cGVHD with prominent fibrotic features treated with belumosudil. We report responses by National Institutes of Health (NIH) organ criteria alongside patient‐reported outcomes and infection‐related healthcare utilization to illustrate patterns of symptomatic and clinical benefit when early organ‐score changes are modest.

## 2 Materials and Methods

This single‐center case report describes two patients who received belumosudil in South Korea. Transplant characteristics, including donor type, HLA and ABO compatibility, stem‐cell source, conditioning regimen, GVHD prophylaxis, and infused cell doses, were abstracted from the medical record. Prior systemic cGVHD therapies and their timing were also reviewed. Clinical events were described primarily in relation to time from allo‐HSCT or cGVHD diagnosis to improve interpretability of the longitudinal clinical course. Belumosudil was prescribed at 200 mg once daily, with background low‐dose corticosteroids at the treating physician's discretion. Overall and organ‐specific responses were evaluated at 3 and 6 months after initiation using NIH organ‐specific criteria. Patient‐reported symptoms were assessed with the modified Lee Symptom Scale (mLSS) at the same time points, and infection‐related hospitalizations were abstracted from the medical record. Safety monitoring consisted of clinical assessment and routine laboratory testing at each visit; adverse events were recorded in the medical record. The report was conducted in accordance with the Declaration of Helsinki. Written informed consent for treatment and publication of de‐identified data was obtained from both patients; per institutional policy, formal Institutional Review Board review was not required for de‐identified, single‐center case reports.

## 3 Case Presentations

### 3.1 Patient 1

Patient 1 was a 35‐year‐old man with acute myeloid leukemia in first complete remission who underwent HLA‐matched, ABO‐matched sibling peripheral blood allo‐HSCT after myeloablative conditioning. Acute GVHD developed approximately 2 months after transplantation, followed by chronic GVHD (cGVHD) approximately 3 months after transplantation, diagnosed and graded according to the 2014 National Institutes of Health (NIH) consensus criteria. Because of late graft failure, he underwent a second allo‐HSCT approximately 77 months after the first transplantation, using HLA‐matched, ABO‐matched unrelated peripheral blood stem cells after reduced‐intensity conditioning. Detailed transplant characteristics, including conditioning regimens, GVHD prophylaxis, and infused cell doses, are summarized in Table S1.

At the time of belumosudil initiation, he had long‐standing severe cGVHD involving the skin, mouth, eyes, lungs, and joints/fascia. The NIH global cGVHD severity was severe, with organ scores of skin, 3; joints/fascia, 3; lungs, 2; mouth, 1; and eyes, 1 (Table 1).

**TABLE 1:** NIH chronic GVHD organ involvement and response to belumosudil in two patients with long‐standing chronic GVHD.

Patient	Time point	NIH organ‐specific score (0–3)^a^										NIH global cGVHD severity^b^	Overall response^c^
		Skin		Mouth	Eye	GI	Liver	Lung		Joint and fascia	Genital		
		BSA	Sclerosis					Symptoms	FEV_1_				
Patient 1	Baseline	3	3	1	1	0	0	2	2	3	0	Severe	
	3 months	3	3	1	1	0	0	2	2	3	0		Stable disease
	6 months	3	3	1	1	0	0	2	2	3	0		Stable disease
Patient 2	Baseline	0	0	0	1	0	0	2	3	0	0	Severe	
	3 months	0	0	0	1	0	0	2	2	0	0		Partial response
	6 months	0	0	0	1	0	0	2	2	0	0		Partial response

Abbreviations: BSA, body surface area; FEV1, forced expiratory volume in 1 s; GI, gastrointestinal; NIH, National Institutes of Health.

^a^ Organ involvement was assessed using the 2014 NIH consensus criteria for chronic GVHD (organ scores 0–3, higher scores indicate greater severity). For skin and lung, component scores used for NIH grading (skin: %BSA and sclerosis; lung: symptom score and FEV1 score) are shown.

^b^ NIH global severity was categorized as mild, moderate, or severe.

^c^ Overall response was determined using NIH response criteria (complete response, partial response, stable disease, or progressive disease).

Before belumosudil, he had received multiple systemic therapies for cGVHD over more than 10 years. Initial treatment after cGVHD onset consisted of cyclosporine‐based therapy with corticosteroids, followed by a short course of mycophenolate mofetil during early refractory disease. He subsequently received cyclophosphamide for persistent manifestations. After the second transplantation, tacrolimus‐based therapy was administered for an extended period. In the later phase of disease, he was treated with ruxolitinib and subsequently imatinib for refractory fibrotic cGVHD. The longitudinal sequence of systemic therapies is shown in Figure 3A.

Belumosudil (200 mg once daily) was initiated more than 11 years after cGVHD diagnosis, on November 18, 2024, with low‐dose prednisolone and continued through this report. No dose modifications or treatment interruptions occurred during the 6‐month observation. Overall response by NIH organ‐specific criteria was stable at 3 and 6 months. Organ scores for the skin, mouth, eyes, lungs, and joints/fascia were unchanged at both time points (Table 1).

**FIGURE 1:**
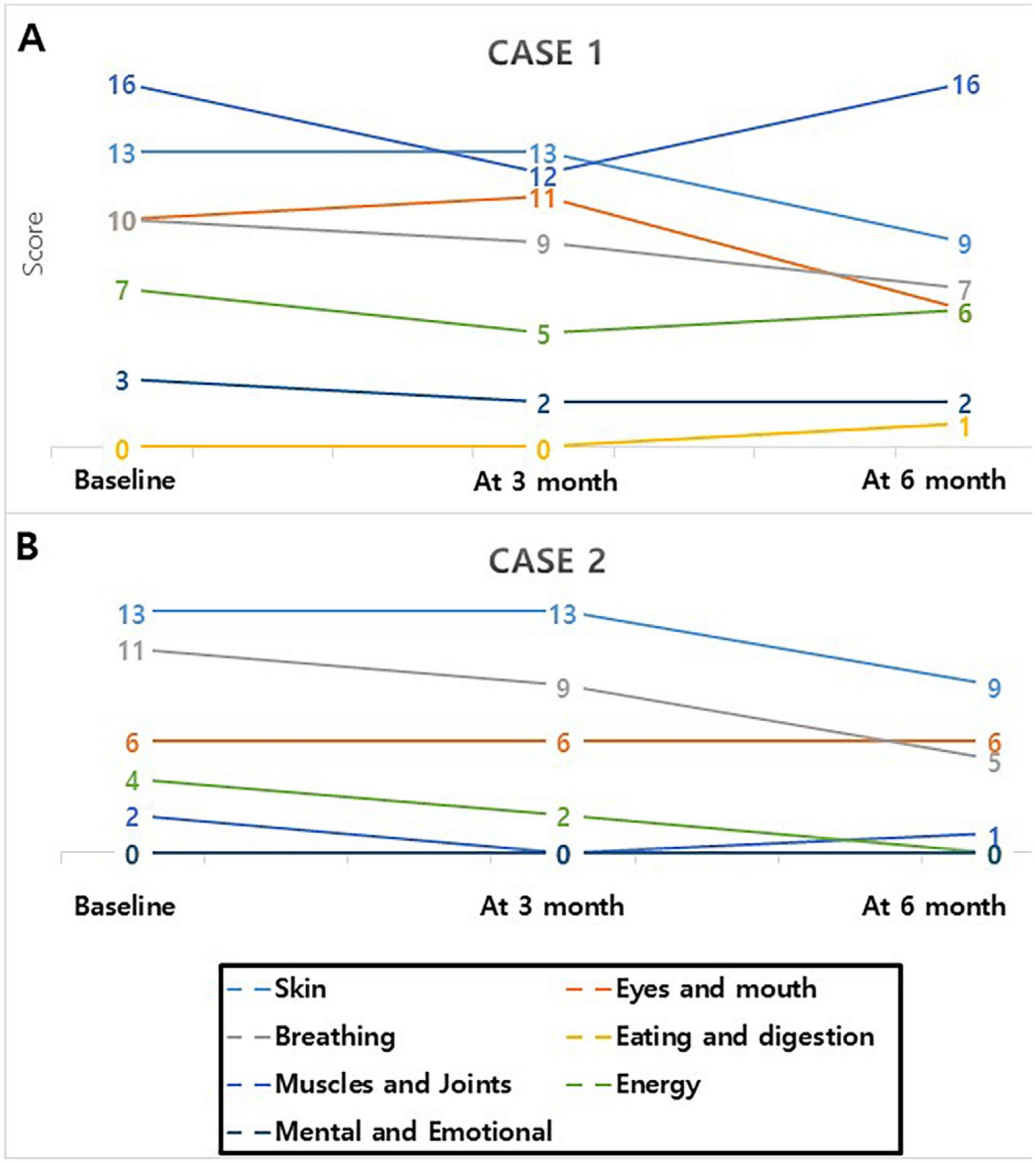
Patient‐reported symptom trajectories measured by the modified Lee Symptom Scale (mLSS) during belumosudil therapy at baseline (pre‐belumosudil), 3 months, and 6 months. (A) Patient 1; (B) Patient 2. Lines depict domain scores; higher scores indicate greater symptom burden.

Patient‐reported symptoms improved. The mLSS total score decreased from 59 at baseline to 52 at 3 months and 47 at 6 months, a 12‐point reduction from baseline. At 6 months, domain scores decreased by 4 points each in the skin, eyes, and mouth, as well as by 3 points in breathing. Among other domains, muscles/joints improved at 3 months but returned to baseline by 6 months; energy and mental/emotional showed small net gains; and eating/digestion rose slightly (Figure 1).

**FIGURE 2:**
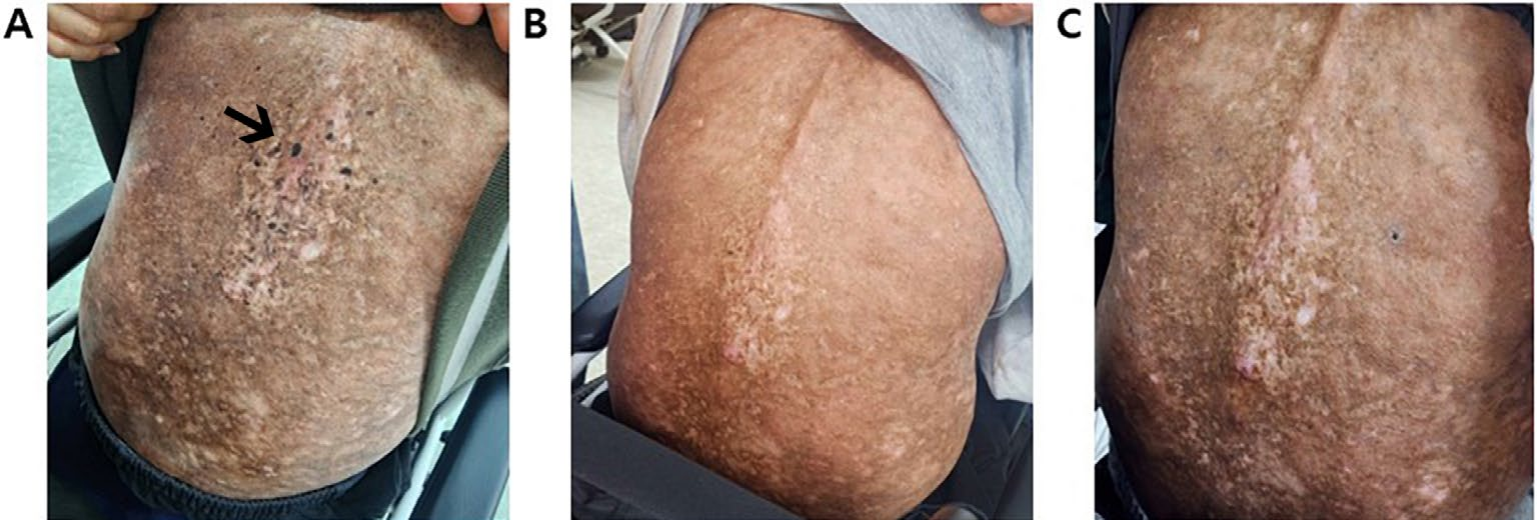
Serial clinical photographs of Patient 1 showing the distribution of sclerodermatous chronic graft‐versus‐host disease (cGVHD) on the posterior trunk at (A) baseline, (B) 3 months, and (C) 6 months. The baseline superficial ulceration (arrows) shows interval healing, and deep sclerotic plaques are less prominent by 6 months.

Clinically, recurrent lower‐limb cellulitis in the setting of sclerodermatous cGVHD with dermal fibrosis and lymphatic dysfunction resolved after belumosudil. He had three cellulitis‐related hospitalizations in the 12 months before therapy and none after initiation through follow‐up, and clinic visit intervals were extended from every 2 weeks to every 8 weeks. Serial photographs of the posterior trunk (Figure 2A–C) showed interval healing of the baseline ulceration and less prominent deep sclerotic plaques with decreased induration and erythema by 6 months, although the NIH skin score remained 3 at all assessments. No adverse events attributable to belumosudil were observed.

Patient Perspective: “Belumosudil has softened my skin and made everyday tasks easier, with no side effects that I can discern.”

### 3.2 Patient 2

Patient 2 was a 48‐year‐old man with myelodysplastic syndrome who underwent allo‐HSCT using HLA‐matched sibling peripheral blood stem cells with major ABO mismatch. He received myeloablative conditioning with fludarabine and busulfan, and GVHD prophylaxis consisted of cyclosporine and methotrexate. Detailed transplant characteristics are provided in Table S1.

He was diagnosed with cGVHD approximately 17 months after allo‐HSCT according to the 2014 NIH consensus criteria. At the time of belumosudil initiation, involved organs included the eyes and lungs. The NIH global cGVHD severity was severe, primarily because of pulmonary involvement (Table 1).

**FIGURE 3:**
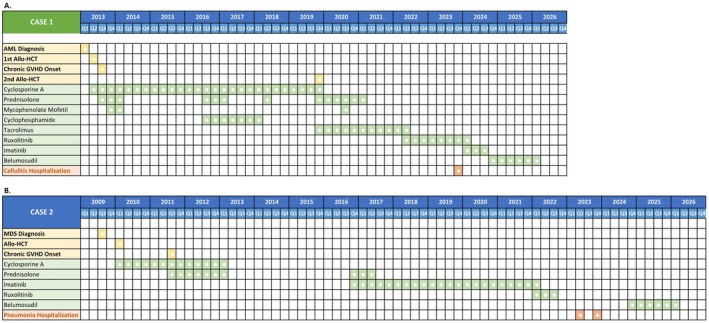
Longitudinal timelines of key events, systemic therapies, and infection‐related hospitalizations. (A) Case 1; (B) Case 2. Allo‐HSCT, allogeneic hematopoietic stem cell transplantation; AML, acute myeloid leukemia; MDS, myelodysplastic syndrome.

Before belumosudil, he had received multiple systemic therapies for cGVHD over more than 10 years. Initial treatment after cGVHD onset consisted of cyclosporine‐based therapy with corticosteroids. Because of persistent fibrotic manifestations, imatinib was introduced in the later phase of disease and continued for an extended period. He subsequently received ruxolitinib for refractory cGVHD before switching to belumosudil. The longitudinal treatment course is summarized in Figure 3B.

Belumosudil (200 mg once daily) was started more than 13 years after cGVHD diagnosis, on November 18, 2024, with prednisolone 5 mg daily and was ongoing at the time of this report. As summarized in Table 1, the lung domain improved at 3 months and was maintained at 6 months, meeting partial response by NIH criteria, while the eye domain remained stable, and other organs showed no worsening; the NIH global cGVHD severity remained severe (Table 1). In parallel, Figure 1B shows a steady decline in total mLSS (23 to 17 to 12), driven predominantly by the breathing domain (11 to 9 to 5). Additional domain trends included decreased skin symptoms (13 to 13 to 9), improved energy (4 to 2 to 0), and a transient improvement in muscles/joints (2 to 0 to 1) that remained better than baseline at 6 months; eyes and mouth were unchanged (approximately 6 at all time points), and eating/digestion and mental/emotional scores were minimal or stable throughout.

In the 12 months before belumosudil, he had two pneumonia‐related hospitalizations. After initiation, no additional pneumonia‐related or other infection‐related hospitalizations occurred during follow‐up. No adverse events attributable to belumosudil were observed.

Patient perspective: “With my breathing easier on belumosudil, I'm able to walk farther with fewer limitations.”

## 4 Discussion

In patients with long‐standing, fibrotic‐predominant cGVHD—as in this series with disease duration greater than 10 years—reversal of established fibrosis is often protracted [1, 4, 5, 6].

Early improvement in patient‐rated symptoms and fewer infection‐related hospitalizations suggest that belumosudil can yield clinically meaningful benefits even when fibrosis is advanced. This aligns with the mechanism of ROCK2 inhibition, which targets both immune dysregulation and profibrotic pathways and therefore may improve patient experience before structural change is captured on clinician‐rated scales [11].

A practical consideration from this experience is the temporal lag between patient‐reported change and organ‐level response by NIH criteria. At early assessments (3–6 months), clinician‐rated NIH organ scores may show stable disease or partial response while patient‐reported outcomes improve. In fibrotic phenotypes, it may therefore be reasonable to continue therapy beyond the initial 3–6‐month evaluations when three conditions are met: patient‐centered improvement is evident, safety is acceptable, and infection‐related morbidity is not worsening. This approach acknowledges that organ‐level change in established fibrosis can lag behind symptomatic benefit.

For chronic lung cGVHD, where therapeutic options remain limited, the observed lung‐domain improvement accompanied by better respiratory symptoms and the absence of pneumonia‐related admissions in one patient is directionally consistent with the expected profile of ROCK2 inhibition [4, 5]. Prospective data in previously treated cGVHD, including analyses focusing on pulmonary involvement, have reported lung‐specific responses with belumosudil [8, 9, 12, 13, 14], supporting its consideration after multiple lines in airway‐predominant disease when early clinical signals emerge. In practice, pairing NIH lung scoring with standard physiologic and functional measures (e.g., serial forced expiratory volume in 1 s, 6‐min walk distance) can help clarify trajectory while therapy is continued.

In parallel, the reduction of cellulitis‐related hospitalizations in a patient with sclerodermatous involvement and lymphatic dysfunction—together with qualitative improvement on serial posterior‐trunk photographs—illustrates how mitigation of fibrosis‐related complications may translate into tangible gains in daily function and healthcare utilization, even when categorical organ scores remain unchanged. Such observations are consistent with a dual immunomodulatory/antifibrotic treatment concept, while stopping short of causal inference.

Tolerability was favorable in both patients and aligns with the published safety experience for belumosudil in previously treated cGVHD [8, 9, 12, 13]. This is clinically relevant in long‐standing disease, where cumulative steroid exposure and multi‐line immunosuppression constrain subsequent choices.

This report has several limitations. It describes two patients with limited follow‐up, which may be insufficient to characterize the therapeutic potential and long‐term safety profile of belumosudil. Histopathology and advanced imaging were not incorporated to evaluate fibrosis dynamics, and causality or generalizability cannot be established. Extracorporeal photopheresis (ECP), an established option for steroid‐refractory or steroid‐dependent cGVHD [15, 16], was not used because it was not available at our institution during the relevant treatment period; therefore, these findings should be interpreted in the context of limited treatment availability.

Despite these limitations, in two patients with long‐standing, fibrotic cGVHD treated under real‐world conditions, belumosudil improved patient‐reported symptoms and reduced infection‐related morbidity, while NIH organ scores were stable or partially improved at early assessments. These observations support continued evaluation and careful monitoring—rather than early discontinuation—when patient‐level benefit is evident and safety is acceptable. Given its ROCK2‐mediated immunomodulatory and antifibrotic mechanism and the possibility of delayed organ response in established fibrosis, belumosudil may be a reasonable option for advanced, fibrotic cGVHD and warrants investigation in larger, prospective cohorts to define the timing, magnitude, and durability of benefit.

## Author Contributions

**Mihee Kim:** visualization, writing – original draft, writing – review and editing. **Jae‐Sook Ahn:** conceptualization, funding acquisition, project administration, supervision, writing – review and editing. **Ho Cheol Jang:** data curation. **Ga‐Young Song:** investigation. **Sung‐Hoon Jung:** investigation. **Seo‐Yeon Ahn:** data curation.

## Funding

This work was supported by the Korea Health Technology R&D Project through the Korea Health Industry Development Institute (KHIDI), funded by the Ministry of Health and Welfare, Republic of Korea (grant numbers RS‐2025‐24536036, RS‐2025‐19252970, and RS‐2024‐00512909). Additional support was provided by the Basic Science Research Program through the National R&D Program for Cancer Control, Ministry of Health and Welfare, Republic of Korea (1720160). A grant (HCRI24037) from the National Research Foundation of Korea (NRF), funded by the Ministry of Science and ICT (MSIT), Republic of Korea (grant numbers 2018R1A2A1A05078480 and 2022R1F1A1063836).

## Consent

Written informed consent was obtained from both patients for publication of this case series and any accompanying images.

## Conflicts of Interest

The authors declare no conflicts of interest.

## Supporting information


**Table S1:** Detailed transplant characteristics of the two patients.

## Data Availability

Data supporting this study are available from the corresponding author upon reasonable request.
