# Supplementary material for: Belumosudil for Long‐Standing Refractory Chronic Graft‐Versus‐Host Disease: A Two‐Patient Case Report
Source: Clin Case Rep. 2026 Jun 25;14(7):e73050. doi: 10.1002/ccr3.73050 (PMC13304232; doi:10.1002/ccr3.73050)
Supplement: Supplementary file 1 — Table S1: Detailed transplant characteristics of the two patients. [file CCR3-14-e73050-s001.docx]

**Supplementary Table S1. Detailed transplant characteristics of the two patients.**

|  | Patient 1 | | Patient 2 |
| --- | --- | --- | --- |
| Characteristic | First Allo-HSCT | Second Allo-HSCT | Allo-HSCT |
| Underlying disease | AML in 1^st^ complete remission | Late graft failure after 1^st^ allo-HSCT | Myelodysplastic syndrome |
| Donor type | Sibling donor | Unrelated donor | Sibling donor |
| Stem-cell source | Peripheral blood stem cells | Peripheral blood stem cells | Peripheral blood stem cells |
| HLA matching | Matched (8/8) | Matched (8/8) | Matched (8/8) |
| ABO compatibility | Matched | Matched | Major mismatch, donor A+ to recipient O+ |
| Conditioning intensity | Myeloablative conditioning | Reduced-intensity conditioning | \| Myeloablative conditioning \| \| --- \| |
| Conditioning regimen | Fludarabine 30 mg/m²/day from day -7 to day -1, and busulfan 3.2 mg/kg/day from -7 to day -4 | Fludarabine 30 mg/m²/day and cyclophosphamide 300 mg/m²/day from day −5 to day −2 | Fludarabine 30 mg/m²/day from day -7 to day -1, and busulfan 3.2 mg/kg/day from -7 to day -4 |
| GVHD prophylaxis | Cyclosporine and methotrexate | Antithymocyte globulin 2.5 mg/kg/day from day −3 to day −1, Tacrolimus | Cyclosporine and methotrexate |
| Infused CD34+ cell dose (×10^6^/kg) | 3.98 | 3.39 | 4.17 |
| Total T-cell dose (×10^7^/kg) | 26.49 | 17.45 | 19.95 |

**Footnotes**

Conditioning regimens and graft-versus-host disease (GVHD) prophylaxis are described according to the transplant protocols used at the time of each allogeneic hematopoietic stem-cell transplantation (allo-HSCT).

**Abbreviations** : allo-HSCT, allogeneic hematopoietic stem-cell transplantation; AML, acute myeloid leukemia; GVHD, graft-versus-host disease; HLA, human leukocyte antigen.
